# Ougan (*Citrus reticulata* cv. *Suavissima*) flavedo extract suppresses cancer motility by interfering with epithelial-to-mesenchymal transition in SKOV3 cells

**DOI:** 10.1186/s13020-015-0042-0

**Published:** 2015-06-27

**Authors:** Linlin Chang, Sheng Jia, Yingying Fu, Tianyi Zhou, Ji Cao, Qiaojun He, Bo Yang, Xian Li, Chongde Sun, Dan Su, Hong Zhu, Kunsong Chen

**Affiliations:** Zhejiang Province Key Laboratory of Anti-Cancer Drug Research, College of Pharmaceutical Sciences, Zhejiang University, Hangzhou, China; Laboratory of Fruit Quality Biology/The State Agriculture Ministry Laboratory of Horticultural Plant Growth, Development and Quality Improvement, Zhejiang University, Hangzhou, China; Zhejiang Cancer Hospital, Hangzhou, China

## Abstract

**Background:**

Ougan (*Citrus reticulata* cv. *Suavissima*) flavedo extract (OFE) exhibited potential anti-tumor effects with unclear underlying mechanisms. This study aims to evaluate the potential anti-metastatic activities of OFE on human ovarian cancer cells, and investigate its inhibitory effect on epithelial-to-mesenchymal transition (EMT).

**Methods:**

Ougan fruits were harvested. Flavedo tissues were separated and made into freeze-dried powder. Then OFE were extracted from the powder. The components of OFE were identified by the high performance liquid chromatography system with a detection wavelength of 280 nm for flavanones and 330 nm for polymethoxylated flavones. Cell viability was assessed by Sulforhodamine B assay. The effects on cancer cell migration and motility were evaluated by wound-healing and transwell assays. The mechanisms of action were investigated by examining the modulation by OFE on EMT-related signaling pathways at the concentrations of 4 μg/mL and 20 μg/mL, using qRT-PCR and western blot analyses.

**Results:**

Non-cytotoxic concentrations of OFE significantly suppressed the cellular migration (4 μg/mL, *P* = 0.005 vs. control group; 20 μg/mL, *P* = 0.003 vs. control group) and motility (4 μg/mL, *P* < 0.001 vs. control group; 20 μg/mL, *P* < 0.001 vs. control group) of SKOV3 cells, and inhibited transforming growth factor-β1 (TGF-β1)-induced E-cadherin loss (4 μg/mL, *P* = 0.002 vs. control group; 20 μg/mL, *P* = 0.001 vs. control group) and mesenchymal marker upregulation, e.g., N-cadherin (4 μg/mL, *P* = 0.027 vs. control group; 20 μg/mL, *P* = 0.013 vs. control group), vimentin (4 μg/mL, *P* = 0.036 vs. control group; 20 μg/mL, *P* = 0.015 vs. control group) and fibronectin (4 μg/mL, *P* < 0.001 vs. control group; 20 μg/mL, *P* < 0.001 vs. control group).

**Conclusions:**

The anti-metastatic ability of OFE inhibited EMT by interfering with the canonical TGF-β1-SMAD-Snail/Slug axis.

## Background

Seventy percents of ovarian cancer patients at their first diagnosis were found to have ascites or distant metastasis. Ovarian cancer has the highest fatality rate among gynecologic malignancies [1]. Accordingly, the development of novel therapeutic agents to prevent or suppress ovarian cancer metastasis, has been considered as a potential strategy to improve the therapeutic effect of ovarian cancer patients.

The epithelial-to-mesenchymal changes in cell phenotype has been defined as the epithelial–mesenchymal transition (EMT), which plays a critical role in cancer metastasis [2, 3]. EMT converts polarized and immotile epithelial cells to motile mesenchymal cells, accompanied by loss of cell–cell adhesion, planar and apical-basal polarity, and acquired mesenchymal features, including motility, invasiveness and enhanced resistance to apoptosis [4]. Blockage of EMT might prevent metastasis at the early stage [5]. Natural products, e.g., Procyanidin C1, Rubus idaeus L, Silibinin, Withania somnifera root extract, Phenolic secoiridoids, have been tested for anti-EMT activity in experimental models [6–10].

Ougan (*Citrus reticulata* cv. *Suavissima*) has exhibited the wide range of biological activities. For instance, extracts from edible tissues of citrus fruit of different species were examined for their antioxidant capacities [11, 12], glucose consumption activities [13–15], and anti-atherosclerosis activities [16, 17]. The anticancer activities of these extracts were exerted through inducing p53-dependent apoptosis [18, 19] and anti-angiogenesis [20]. Currently, no studies have investigated the effects of Ougan flavedo extract (OFE) on EMT.

This study aims to evaluate the potential anti-metastatic activities of OFE on human ovarian cancer cells, and investigate its inhibitory effect on EMT.

## Methods

### Preparation of OFE

**Fig. 1:**
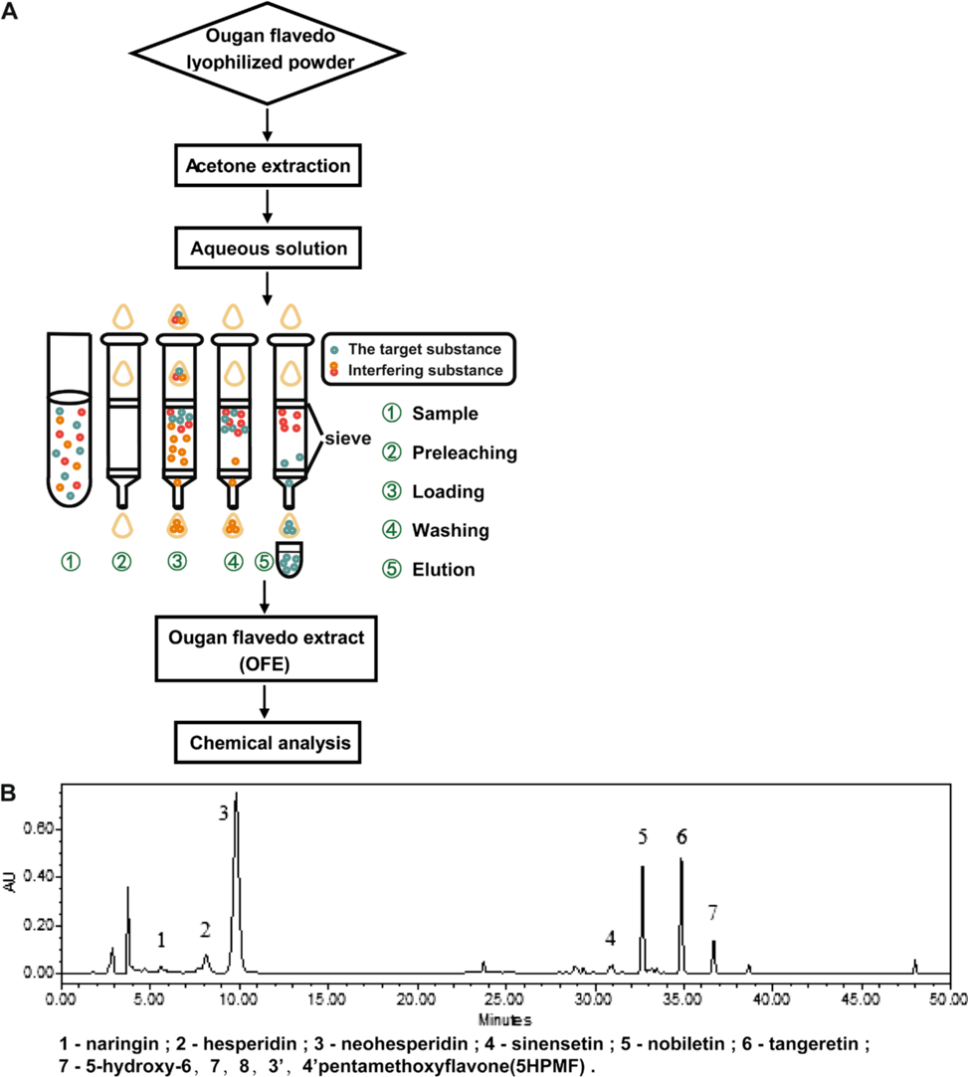
Extraction and identification of OFE. **a** Preparation procedure of OFE. **b** HPLC profile of OFE (λ = 280 nm)

Ougan fruits were harvested from Ouhai District of Wenzhou, Zhejiang province, China in 2011. The fruit samples were botanically authenticated by Dr. Changjie Xu from Zhejiang University. Flavedo tissues of Ougan fruit were manually separated. They were frozen in liquid nitrogen and then stored at −80 °C. After freeze-drying (FM 25EL-85, VirTis, USA), they were ground into a fine powder and stored at −80 °C until extraction and analysis. Lyophilized Ougan flavedo powder (1 g) was dissolved in 20 mL of 80 % acetone (solid–liquid ratio 1:20) and then sonicated (JBT/C-YCL500T/3P, Jinbaite Electronic Co., Ltd, Jining, China) for 30 min, with a frequency of 60 kHz and a power of 30 W (Fig. 1a). The extract was centrifuged (Centrifuge 5804 R, Eppendorf, Germany) at 1932 × *g* for 10 min. The residue was extracted twice as described above, and the supernatants were combined and evaporated on a rotary evaporator (Heidolph, LABOROTA 4000-efficient, Germany), and the residue was dissolved in 10 mL of deionized water. Samples were then purified by a C18 Sep-Pak Cartridge column (12 cc/2 g, Waters, Milford, MA, USA). First, the column was activated by 20 mL of methanol, and balanced with 20 mL of deionized water. The aqueous solution was loaded at a rate of 1 mL/min. After the column was fully adsorbed, it was washed with 40 mL of deionized water to remove the sugar, acid and other polar substances in the flavedo. The column was eluted with 40–80 mL of methanol (depending on the eluting color) and the eluent was evaporated into powder; this residue was OFE and was analyzed by the high performance liquid chromatography (HPLC) system. OFE was used for the bioactivity assay. Naringin, hesperidin, neohesperidin, naringenin, hesperetin, and acetonitrile were purchased from Sigma-Aldrich (St. Louis, MO, USA). Sinensetin was purchased from Hengdailao, Co., Ltd. (Shanghai, China). Nobiletin and tangeretin were the products of J&K Scientific Ltd. (Shanghai, China). 5-Hydroxy-6,7,8,3’,4’-pentamethoxyflavone (5HPMF) was purchased from Feiyu Bio tech Co., Ltd. (Nantong, China).

### HPLC analysis

Flavonoid compounds in OFE were analyzed by HPLC according to Sun et al. [20] with some modifications. An HPLC system (2695 pump, 2996 diode array detector, Waters, Milford, MA, USA) coupled with an ODS C18 analytical column (Waters, Sunfire, 4.6 × 250 mm, i.d., 5 μm, Milford, MA, USA) was used with a detection wavelength of 280 nm for flavanones and 330 nm for polymethoxylated flavones. The mobile phase of HPLC consisting of deionized water (A) and acetonitrile (B) was performed as follows: 0–15 min, 20 % B; 15–35 min, 20–60 % B; 35–40 min, 60–100 % B; 40–42 min, 100 % B; 42–45 min, 10–20 % B; 45–50 min, 20 % B. The separation temperature was set at 25 °C and the flow rate was 1 mL/min.

### Cell culture

Human ovarian cancer cell line SKOV-3 was purchased from Shanghai Institute of Biochemistry and Cell Biology, Chinese Academy of Medical Sciences (TCHu185, Shanghai, China). SKOV3 cells were maintained in RPMI-1640 medium (Gibco, Grand Island, NY, USA) supplemented with 10 % FBS (Gibco, Grand Island, NY, USA) and cultured in a humidified atmosphere of 95 % air plus 5 % CO_2_ at 37 °C. Cells were plated the day before to induce EMT. After starvation in serum-free medium overnight, 5 ng/mL TGF-β1 was added; cells were examined after treatment for 16 h.

### Cell viability

SKOV3 cell viability after OFE exposure was evaluated by Sulforhodamine B (SRB) assay. After the incubation period, cell monolayers were fixed with 10 % (wt/vol) trichloroacetic acid and stained for 1 h at 4 °C. The plates were washed five times with deionized water, and then dried in an oven at 60 °C. When completely dry, 50 μL of SRB was added to each well for 20 min, and then the excess dye was removed by washing five times with 1 % (vol/vol) acetic acid, followed by further drying in an oven at 60 °C. The protein-bound dye was dissolved in 10 mM Tris-base solution for OD determination at 510 nm by a Multiskan Spectrum (Thermo Electron Corporation, Marietta, OH, USA). Cell viability was calculated for each well as [OD510 treated cells/OD510 control cells] × 100 %.

### Wound-healing assay

Cells were plated in 24-well culture plates in complete culture medium and grown to confluence. A wound was made by scraping the middle of the cell monolayer with a sterilized 200-μL pipette tip. Cells were then cultured with fresh complete culture medium containing 10 % FBS with OFE or the vehicle (0.2 % DMSO). Then, the ability of cells to migrate into the cleared section was observed and photographed by a microscope (Leica DMI 4000 B, Wetzlar, Germany).

### Transwell assay

A Boyden chamber system (Costar Corp., Cambridge, MA, USA) was used for transwell migration assays. A total of 3 × 10^4^ SKOV3 cells (serum-starved overnight) were seeded into each insert in serum-free media, while serum with or without OFE treatment was placed in the wells below. After incubation for 24 h, cells remaining in the top of the inserts were removed using a cotton swab. The cells that had migrated through the filter were fixed with 75 % ethanol for 30 min followed by 0.1 % crystal violet staining for 20 min. The ability of cells to migrate to the lower chamber was observed and photographed by microscopy (Leica DMI 4000 B, Wetzlar, Germany). Then an equal volume of 10 % acetic acid was added to each well to completely dissolve the stained crystal violet. OD570 was read by a Multiskan Spectrum (Thermo Electron Corporation, Marietta, OH, USA) to quantify the percentage of migrated cells.

### Western blot analysis

Protein samples were size-fractionated by SDS-PAGE and transferred to PVDF membranes (Millipore, Bedford, UK). Blots were blocked for 1 h in 5 % milk/0.1 % Tween 20 in phosphate buffered saline (PBS-T) and then incubated with primary antibodies (1: 1000) at 4 °C overnight. Blots were then washed three times for 15 min in PBS-T, followed by incubation with secondary antibody (according to different primary antibodies, HRP-conjugated goat anti-mouse, anti-rabbit, and rabbit anti-goat IgG were used (1: 5000, Santa Cruz, Dallas, TX)) in 5 % milk/PBS-T for 1 h, and then washed three times for 15 min in PBS-T. The membranes were briefly incubated with ECL detection reagent (Amersham Biosciences, Castle Hill, Australia) to visualize the proteins and were then exposed on X-ray film. Primary antibodies used were as follows: anti-E-cadherin (Cat# 610405), anti-N-cadherin (Cat# 610921) and Fibronectin (Cat# 610078) were purchased from BD; anti-Vimentin (Cat# ab8069) was from Abcam Ltd. (Cambridge, UK); anti-Slug (Cat# 9585), anti-Smad2/3 (Cat# 3102), anti-p-Smad2 (Cat# 3108), and anti-p-Smad3 (Cat# 9520) were from Cell Signaling Technology (Beverly, MA); anti-Snail (Cat# AF3639) was from R&D (Minneapolis, MN); anti-β-Actin (Cat# Sc-1615) was from Santa Cruz (Dallas, Texas, US).

### Real-time PCR

SKOV3 cells were treated with vehicle or OFE, followed by TGF-β1. Subsequently, total RNA was prepared using the EasyPure RNA Kit (Cat# H30828; TransGen Biotech, Beijing, China). Single-strand cDNA was prepared from the purified RNA using oligo (dT) priming (Thermoscript RT kit; Invitrogen, Carlsbad, CA, USA), followed by SYBR-Green real-time PCR (Qiagen, Hilden, Germany). The sequences of oligonucleotide primers were as follows: *FN1* (forward primer: 5′-TCCAAGCGGAGAGAGT-3′; reverse primer: 5′-GTGGGTGTGACCTGAG-3′), *SNAI2* (forward primer: 5′-AGATGCATATTCGGACCCAC-3′; reverse primer: 5′-CCTCATGTTTGTGCAGGAGA-3′), *SNAI1* (forward primer: 5′-GAAAGGCCTTCAACTGCAAA-3′; reverse primer: 5′-TGACATCTGAGTGGGTCTGG-3′), *GAPDH* (forward primer: 5′-GTCATCCATGACAACTTTGG-3′; reverse primer: 5’-GAGCTTGACAAAGTGGTCGT-3’) and were synthesized by Sangon (Shanghai, China). Reactions of 20-μL volume/well contained 10-μL SYBR Premix Ex Taq (2×) (TaKaRa Biomedical Technology, Otsu, Japan), 0.6-μL forward primer (10 μM), 0.6-μL reverse primer (10 μM), 7.8-μL dH_2_O and 1.0-μL DNA template. RT-PCR reactions were performed according to the manufacturer’s instructions. Reverse transcription and amplification were performed at 95 °C for 15 min, followed by 40 cycles of 95 °C for 5 s and 55 °C for 30 s, and 72 °C for 30 s using an Eppendorf Mastercycler ep Realplex 4 (Eppendorf, Hamburg, Germany), operated by the realplex software (Eppendorf). Data quantitation was performed by the relative standard curve method.

### Statistical analysis

**Fig. 2:**
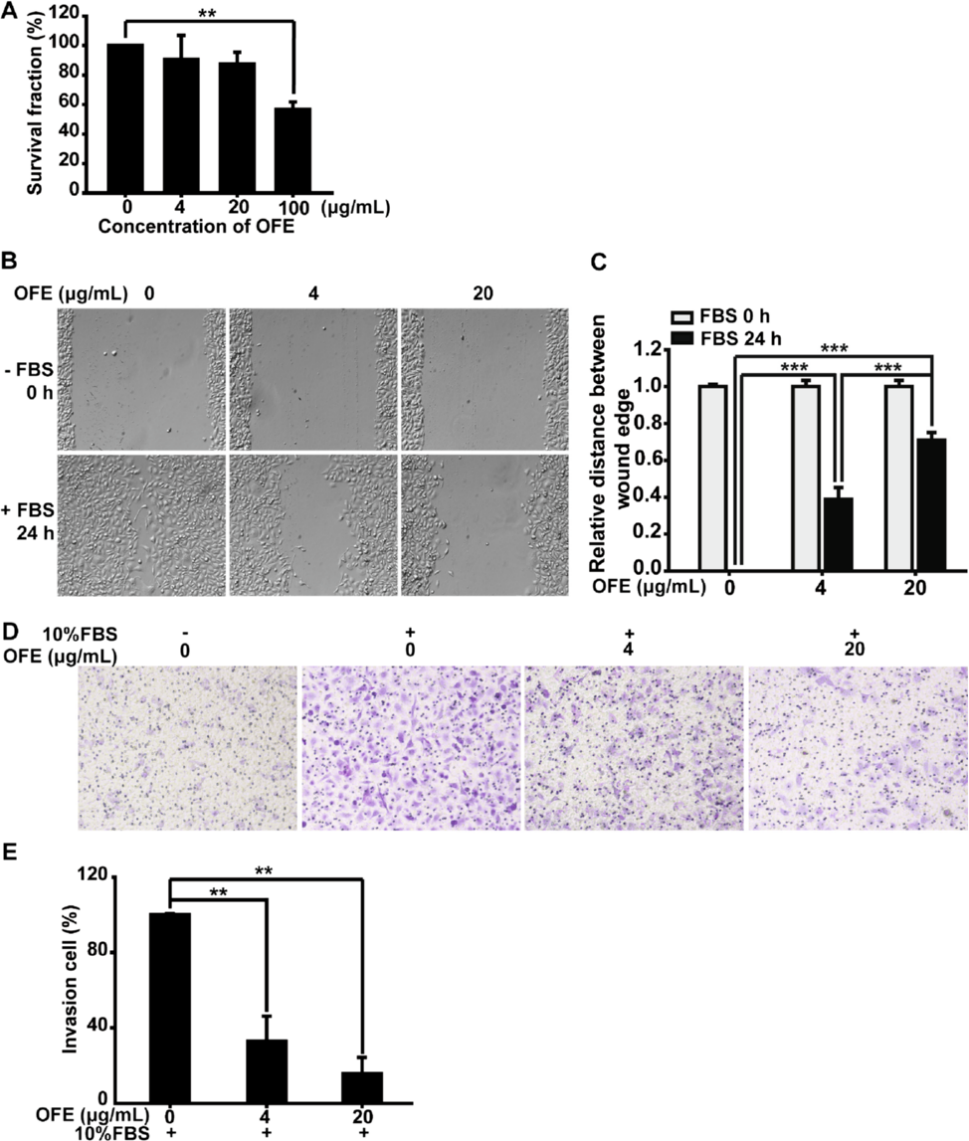
OFE suppressed the motility of cancer cells. **a** SRB assay was performed to assess the viability of SKOV3 cells in the presence of OFE. ** *P < 0.01* vs. control; two-tailed Student’s t-tests. **b** Wound-healing assays were used to determine the effects of OFE or the DMSO vehicle on cancer cell motility. **c** The wound healing results were scored in a double-blind method analyzing several areas of the wound. **d** Transwell assays were also used to determine the effects of OFE or the DMSO vehicle on cancer cell motility. **e** Crystal violet was solubilized with 200-μL 10 % acetic acid per well, and the migratory rate was quantified by measuring the OD. Data are presented as mean ± SD from three independent experiments. ** *P < 0.01* vs. control; ANOVA followed by LSD

Experiments were performed at least three times or as indicated. Data are presented as mean ± SD from three independent experiments. Statistical analyses were performed using ANOVA followed by LSD for comparing multiple groups, and two-tailed Student’s *t*-test was employed for two-group comparisons for Fig. 2a. *P* values less than 0.05 were considered significant (* *P* < 0.05, ** *P* < 0.01 and *** *P* < 0.001). The concentration-dependent manner was visually determined.

## Results

### Identification of main compounds

Characterization of flavonoid profiles in OFE was accomplished by HPLC. Three flavanone glycosides, naringin, hesperidin and neohesperidin, and four polymethoxylated flavones, sinensetin, nobiletin, tangeretin and 5HPMF, were identified according to retention time and UV pattern compared with their standards (Fig. 1b).

### Viability of SKOV3 cells in the presence of OFE

SRB assays were performed to assess the potential effects of OFE on the proliferation of cancer cells. No significant loss of SKOV3 cell viability was observed in the presence of OFE up to 20 μg/mL for 24 h (Fig. 2a). When the concentration of OFE reached 100 μg/mL, cell viability was decreased to approximately 55 % (*P* = 0.003 vs. control group). To exclude the interference of cytotoxicity on cancer cell motility caused by OFE, we investigated the activities of OFE on EMT and cancer cell motility at concentrations of 4 and 20 μg/mL.

### OFE suppressed the motility of SKOV3 cells

Wound-healing and transwell assays were further utilized to determine the cellular migration abilities of SKOV3 cells after treatment with vehicle or OFE.

For wound-healing assays, medium containing 10 % FBS with or without OFE was added after scraping to produce a “wound”. FBS promoted SKOV3 cells to migrate and close the wound, while co-incubation with OFE significantly (4 μg/mL, *P* < 0.001 vs. control group; 20 μg/mL, *P* < 0.001 vs. control group) reversed the migration of SKOV3 cells (Fig. 2b). The results were quantified using a double-blind method by analyzing several areas of the wound (Fig. 2c).

For transwell assays, SKOV3 cells were treated with or without OFE for 24 h; cells that had migrated onto the lower surface of the porous membrane were observed with an inverted microscope after crystal violet staining. The total number of cells on the lower surface was increased much more highly by medium containing 10 % FBS, compared with the group without FBS stimuli (Fig. 2d). OFE treatment (4 and 20 μg/mL) for 24 h significantly suppressed the migration of SKOV3 cells induced by FBS stimuli, as indicated by inhibition ratios of 57.6 % (*P* = 0.005 vs. control group) and 78.2 % (*P* = 0.003 vs. control group), respectively (Fig. 2e).

### OFE prevented TGF-β1-induced downregulation of E-cadherin protein

TGF-β1 could induce EMT [21, 22]. We introduced TGF-β1 to stimulate the onset of EMT, and evaluated the effects of OFE on EMT.

**Fig. 3:**
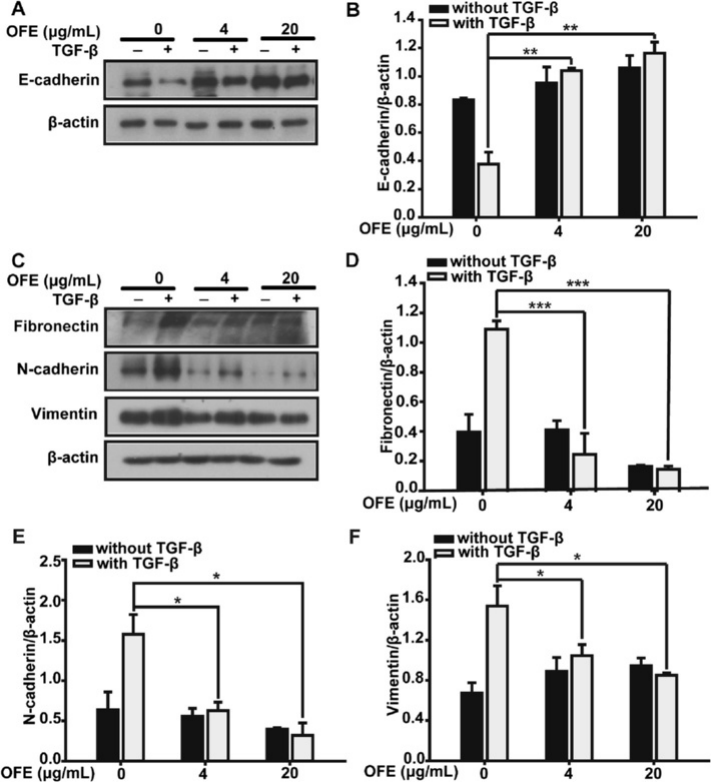
OFE attenuated the loss of E-cadherin and abrogated the increase in mesenchymal markers caused by TGF-β1. **a** SKOV3 cells treated as indicated were assayed by western blot analysis (WB) to assess the expression of epithelial marker E-cadherin. β-actin was used as a loading control. **b** The quantitative ratios are shown as relative optical densities of bands that are normalized to β-actin expression. **c** Mesenchymal markers, including N-cadherin, fibronectin and vimentin, were examined by western blot analysis. (**d**, **e** and **f**) The quantitative ratios are shown as relative optical densities of bands that are normalized to the expression of β-actin. Cells were incubated with TGF-β1 (5 ng/mL) and increasing concentrations of OFE (4 and 20 μg/mL) for 48 h. * *P* < 0.05, ** *P* < 0.01 and *** *P* < 0.001 vs. control; ANOVA (LSD)

Loss of E-cadherin has been suggested as the main marker of EMT occurrence [23]. Herein, we carried out western blot analysis to estimate the protein level changes in SKOV3 cells (Fig. 3a). E-cadherin protein was maintained at a high level in unstimulated SKOV3 cells, but was reduced by approximately 50 % upon TGF-β1 treatment, indicating the transition from an epithelial to a mesenchymal status of TGF-β1-treated cells. However, co-incubation with OFE significantly prohibited the loss of E-cadherin. As the results of semi-quantitative densitometric analyses of western blotting showed, E-cadherin protein levels were increased by approximately 2.5-fold after OFE treatment (Fig. 3b) (4 μg/mL, *P* = 0.002 vs. control group; 20 μg/mL, *P* = 0.001 vs. control group). The level of E-cadherin also increased in unstimulated cells, suggesting that OFE could up-regulate basal E-cadherin levels (Fig. 3a).

Collectively, these data indicated that OFE could inhibit TGF-β1-induced downregulation of E-cadherin in SKOV3 cells.

### OFE attenuated TGF-β1-induced augmentation of mesenchymal markers

Increased expression of mesenchymal markers, e.g., N-cadherin, vimentin and fibronectin, contributes to the enhanced motility of cancer cells. In this study, the inhibitory effect of OFE on EMT was supported by observations of the expression of several mesenchymal markers. In SKOV3 cells, OFE suppressed the upregulation of N-cadherin (4 μg/mL, *P* = 0.027 vs. control group; 20 μg/mL, *P* = 0.013 vs. control group), vimentin (4 μg/mL, *P* = 0.036 vs. control group; 20 μg/mL, *P* = 0.015 vs. control group) and fibronectin (4 μg/mL, *P* < 0.001 vs. control group; 20 μg/mL, *P* < 0.001 vs. control group) triggered by TGF-β1 (Fig. 3c), which could also be observed from semi-quantitative densitometric analyses of western blot results (Fig. 3d, e and f).

**Fig. 4:**
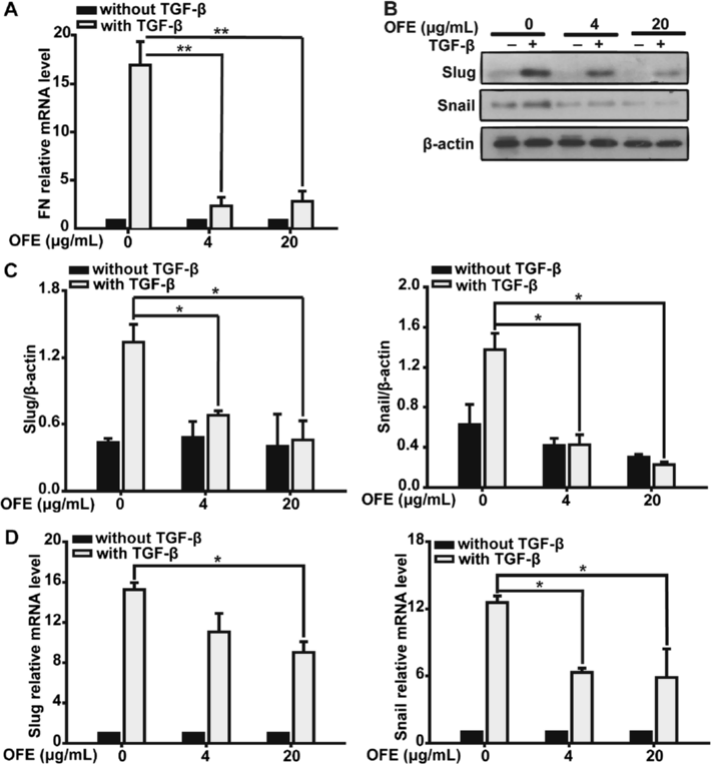
TGF-β1-induced upregulation of Slug protein and mRNA expression was blocked by OFE. **a** TGF-β1-induced upregulation of *FN1* mRNA expression was blocked by OFE. **b** Western blot analysis was performed to examine the expression of Slug and Snail after SKOV3 treatment, as indicated. **c** Protein levels were further confirmed by semi-quantitative densitometry analyses. **d** Real-time PCR analysis to measure the mRNA levels of *SNAI1* and *SNAI2* in SKOV3 cells. Data are presented as mean ± SD from three independent experiments. * *P* < 0.05, ** *P* < 0.01 and *** *P* < 0.001 vs. control; ANOVA (LSD)

To investigate whether OFE transcriptionally regulated the expression of mesenchymal markers, we used quantitative real-time PCR to examine the mRNA levels of fibronectin (*FN1*). Upon TGF-β1 stimulation, *FN1* mRNA levels increased remarkably, while in the OFE-treated groups, the increase caused by TGF-β1 was attenuated (Fig. 4a) (4 μg/mL, *P* = 0.009 vs. control group; 20 μg/mL, *P* = 0.008 vs. control group).

### TGF-β1-induced upregulation of Slug protein and mRNA expression was blocked by OFE

The individual extracellular inducers provoke the expression of EMT-associated transcription factors [24], such as Twist, Snail, Slug or ZEB1, and subsequent activation of the EMT program. The importance of these transcriptional factors in the activation and regulation of EMT has been demonstrated by numerous reports [2–4, 21, 25]; of note is the fact that we observed a remarkable decrease in *FN1* mRNA in OFE-treated cells, raising the possibility that OFE might have a regulatory effect on transcription factors. We investigated the effects of OFE on the expression of Slug and Snail. Distinct augmentation of Slug and Snail protein levels could be observed in TGF-β1-treated SKOV3 cells (Fig. 4b), which was consistent with previous reports [26, 27]. However, such increases in Slug and Snail protein in these cancer cells were remarkably abrogated upon exposure to OFE, which was further confirmed by semi-quantitative densitometry analyses of western blotting results (Fig. 4c).

qRT-PCR analysis was utilized to determine the mRNA levels of Slug (*SNAI2*) and Snail (*SNAI1*) in SKOV3 cells. OFE suppressed the augmentation of *SNAI2* and *SNAI1* mRNA under TGF-β1 stimuli, as indicated by the reduced fold-change from 15.3 to 9.0, and 12.6 to 5.9, respectively (Fig. 4d).

These results indicated that OFE attenuated the elevation of mRNA and protein levels of Slug and Snail caused by TGF-β1, and might result in the subsequent manipulation of EMT-associated factors such as fibronectin, whose mRNA levels were reduced when TGF-β1-stimulated cells were exposed to OFE.

### Treatment of OFE interfered with TGF-β1-activated SMAD pathway

The TGF-β1-induced SMAD signaling cascade is the canonical pathway in EMT [5]. TGF-β1 activates R-SMADs, which form hetero-oligomers with SMAD4 regulating the expression of some transcription factors such as Slug or Snail [28].

**Fig. 5:**
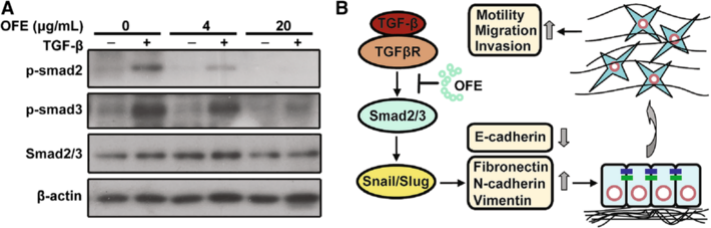
OFE treatment counteracted TGF-β1-activated SMAD pathways. **a** The effect of OFE on TGF-β1-induced phosphorylation of SMAD2 and SMAD3 was investigated by western blot analysis in SKOV3 cells. **b** TGF-β1-induced SMAD pathway activation that resulted in EMT was inhibited by OFE

To clarify the mechanism underlying the inhibitory effect of OFE on TGF-β1 signals, we explored the effect of OFE on TGF-β1-induced phosphorylation of SMAD2 and SMAD3. In SKOV3 cells, TGF-β1 alone induced phosphorylation of SMAD2 and SMAD3, denoting the activation of these two R-SMADs; co-treatment with OFE reduced the levels of p-SMAD2 (4 μg/mL, *P* = 0.002 vs. control group; 20 μg/mL, *P* = 0.001 vs. control group) and p-SMAD3 (4 μg/mL, *P* = 0.035 vs. control group; 20 μg/mL, *P* = 0.004 vs. control group), whereas the total expression of SMAD2/3 remained unchanged (Fig. 5a). OFE modulated critical regulatory steps of the SMAD pathway activated by TGF-β1 in EMT, in particular by abrogating the phosphorylation of R-SMADs, and consequently counteracting the instigation of transcriptional activity of SMAD complexes (Fig. 5b).

## Discussion

Citrus (*Citrus reticulate Blanco*) peel extract could induce apoptosis in prostate tumors in a human prostate tumor xenograft mouse model, which was accompanied by mechanistic downregulation of inflammatory enzymes, including inducible nitric oxide synthase (NOS) and cyclooxygenase-2 (COX-2), factors involved in metastasis (MMP-2 and MMP-9), angiogenesis (vascular endothelial growth factor, VEGF), and proliferative molecules (PCNA) [29]. The citrus fruit extract from *Citrus hassaku Hort. ex Tanaka* suppressed metastasis, probably through inhibition of C-X-C chemokine receptor type 4 (CXCR4) and MMP-9 in MDA-MB-231 cells [30]. However, the extract from *Citrus unshiu Marc* could inhibit MDA-MB-231 cell adhesion to endothelial cells by selective inhibition of VCAM-1, but not MMP-9 expression [31].

In this study, OFE prevented the onset of metastasis. OFE contained several classes of flavonoid compounds chemically characterized as naringin, hesperidin, neohesperidin, poncirin, sinensetin, nobiletin, tangeretin, and 5-hydroxy-6,7,8,3′,4′pentamethoxyflavone (5HPMF) from HPLC analysis. Some of these bioactive chemical constituents exhibited anti-metastasis activities [32–34]. In human breast cancer cells, nobiletin downregulated the constitutive expression of CXCR4 and MMP-9 [32]. In human hepatocellular carcinoma cells, hesperidin inhibited acetaldehyde-induced matrix metalloproteinase-9 gene expression [33]. In addition, tangeretin inhibited the invasion of MO4 cells (Kirsten murine sarcoma virus transformed fetal mouse cells) into embryonic chick heart fragments in vitro [34].

Little is known about the effects of these flavonoid compounds on EMT. In this study we demonstrated for the first time the inhibitory effects of OFE on ovarian cancer metastasis, particularly interference with EMT. We showed that OFE could inhibit TGF-β1-induced EMT through the canonical TGF-β1-SMAD-Snail/Slug axis.

Many growth and differentiation factors, including growth factors [35, 36], Wnt [37] and Notch proteins [38], can induce EMT. TGF-β was first demonstrated to induce EMT in cell culture. In this study, we observed that OFE could interrupt the occurrence of EMT in TGF-β1-treated SKOV3 cells through counteracting E-cadherin loss and fibronectin upregulation. Various studies have explored the roles of TGF-β-activated SMADs in EMT [39–41]. TGF-β binds to its receptors, leading to the activation of SMAD2 and SMAD3 through phosphorylation by TGF-β receptor I. Phosphorylated SMAD2 and SMAD3 form trimers with SMAD4, then translocate to the nucleus where they associate and cooperate with DNA binding transcriptional factors to regulate the expression of TGF-β target genes including *SNAI1* and *SNAI2* to promote EMT [42]. TGF-β receptors, SMAD2, SMAD3 and SMAD4, are all essential for TGF-β-induced EMT, as suppressing the expression of these genes by dominant-negative forms blocks TGF-β-induced EMT [43]. The interruption of EMT by OFE was attributed to the intervention of the SMAD signaling pathway, demonstrated by the attenuated phosphorylation of SMAD2/3 and reduction in the transcriptional activity of the SMAD complexes.

The suppressive effects on cell motility and EMT in SKOV3 cells by OFE suggested the actions of this extract on ovarian cancer metastasis. Most of these effects could be attributed to the presence of several flavonoid compounds in the extract. But it is still unclear whether the anti-EMT effects of the extract were due to individual or combined effects of these components. Further studies to characterize the bioactive chemical constituents and elucidate the mechanism of action of OFE are in progress.

## Conclusions

The anti-metastatic ability of OFE inhibited EMT by interfering with the canonical TGF-β1-SMAD-Snail/Slug axis.

## Abbreviations

OFE: Ougan flavedo extract
EMT: Epithelial-to-mesenchymal transition
HPLC: High performance liquid chromatography
PBS: Phosphate buffered saline
RT-PCR: Real time PCR
TGF-β1: Transforming growth factor-β1
5HPMF: 5-Hydroxy-6,7,8,3′,4′–pentamethoxyflavone
SRB: Sulforhodamine B
FBS: Fetal bovine serum
NOS: Nitric oxide synthase
COX-2: Cyclooxygenase-2
MMP2: Matrix metalloproteinase 2
VEGF: Vascular endothelial growth factor
CXCR4: C-X-C chemokine receptor type 4
